# Precise Control of the Preparation of Proton Exchange Membranes via Direct Electrostatic Deposition

**DOI:** 10.3390/polym14193975

**Published:** 2022-09-23

**Authors:** Hao Liu, Runmin Tian, Chunxu Liu, Jinghan Zhang, Mingwei Tian, Xin Ning, Xingyou Hu, Hang Wang

**Affiliations:** 1 Industrial Research Institute of Nonwovens & Technical Textiles, College of Textiles & Clothing, Qingdao University, Qingdao 266071, China; 2 Shandong Special Nonwoven Materials Engineering Research Center, Qingdao University, Qingdao 266071, China; 3 State Key Laboratory of Bio-Fibers and Eco-Textiles, Qingdao University, Qingdao 266071, China

**Keywords:** direct electrostatic deposition, proton exchange membrane, direct methanol fuel cell, ultrathin membrane, high power density

## Abstract

In this work, we reported a novel preparation method for a proton exchange membrane (PEM) named, the direct electrostatic deposition method. In theory, any required thickness and size of PEM can be precisely controlled via this method. By direct electrostatic spraying of Nafion solution containing amino modified SiO_2_ nanoparticles onto a metal collector, a hybrid membrane of 30 μm thickness was fabricated. The DMFC assembled with a prepared ultrathin membrane showed a maximum power density of 124.01 mW/cm^2^ at 40 °C and 100% RH, which was 95.29% higher than that of Nafion. This membrane formation method provides potential benefits for the preparation of ultrathin PEMs.

## 1. Introduction

The use of fossil fuels brings about tremendous problems for resources and the environment, such as greenhouse effects, acid rain, ozone depletion, etc. [1,2]. Among them, greenhouse effects have caused concern around the world due to their serious effect on the environment and climate. Global decarbonization is of great importance, and China has put forward its carbon-neutral strategy [3]. Therefore, the research and development of new energy conversion devices will be vigorously promoted. However, it is difficult to apply renewable energy sources (such as solar energy and wind energy) continuously and stably due to their instability and intermittence during generation [4]. To tackle this issue, the employment of electrochemical energy storage systems, especially direct methanol fuel cells (DMFCs), has received wide attention throughout the world [5,6,7,8]. In the future, they will play a major role in improving energy efficiency and reducing fossil fuels. In DMFC components, proton exchange membranes (PEMs) act as proton-conductive mediums for protons as well as barriers for the passage of electrons and fuels between the anode and cathode components [9,10,11,12]. PEM is one of the key components which can directly affect the performance of DMFCs. Perfluorinated sulfonic acid resin, such as Nafion from Dupont, has been widely used as the PEM in DMFC because of its excellent chemical stability, and good mechanical strength derived from the hydrophobic PTFE backbone. Furthermore, the ionic domains formed between the hydrophilic–SO_3_H in the side chain and the hydrophobic PTFE backbone in the Nafion structure could provide good proton conductivity (≥0.1 S cm^−1^), which ensures its practical applications [13]. However, some drawbacks, such as high cost, high methanol permeability, and low proton conductivity under low humidity conditions, drastically limit the widespread commercial application of Nafion in fuel cells.

Recently, nanocomposites have raised a lot of research interest in the preparation of PEMs due to the significant improvement in performance based on the nature of nanomaterials, including nanoparticles, nanowires, nanofibers, nanosheets, etc. [14,15]. Among these nanomaterials, silica has attracted the greatest interest due to its high specific surface area and convenient surface modification. Much literature has proved that the addition of inorganic silica to PEM polymers can improve thermal stability, proton conductivity as well as methanol resistance [16]. Zhao et al. [17] prepared composite PEMs by doping amino-functionalized mesoporous silica (AMS) with SP/IL (N-ethylimidazole trifluoromethanesulfonate and found that amino-functionalized mesoporous silica contributed to the proton transfer due to large lumen channels and acid–base pairs between –NH_2_ and –SO_3_H. The prepared composite membrane with AMS reached a high proton conductivity of 1.494 mS/cm under anhydrous conditions at 200 °C, which is four times that of the composite membrane with pure silica. Mahdavi et al. [18] presented a novel nanocomposite PEM containing sulfonated polysulfone, metal–organic frameworks and silica nanoparticles. The combination of silica nanoparticles and MOFs in a matrix can act as proton hopping sites to enhance the transport efficiency of protons. Results showed that the prepared PEMs containing 5% nanoparticles demonstrated a high proton conductivity of 17 mS/cm at 70 °C and a maximum power density of 40.80 mW/cm^2^. These experiments proved that functional silica is of great significance to the performance of PEMs.

Nowadays, the strategies and techniques for the preparation of PEM mainly include recasting or blending [19,20,21], hot-pressing [22,23] and impregnation [24,25,26,27]. Recasting is a simple and low-cost membrane formation method that can offer easy optimization of the processing parameters. The primary requirement for this method is to have the materials well-dissolved in the solvent to ensure the solution is uniform and homogeneous. Hot-pressing is a method for preparing PEMs by means of the difference in melting temperatures of poly-materials. The dense membrane can be prepared by this method only at high temperatures and pressure. Ballengee [28] prepared composite PEMs via hot pressing (127 °C and 15,000 psi) and annealing (from 130 °C to 250 °C). In this process, melted Nafion flowed into the void space between the polyphenylsulfone nanofibers to create a fully dense membrane structure. As the name suggests, the impregnation method refers to incorporating porous materials in a polymer matrix to form a dense membrane [29]. Similar to hot-pressing, the impregnated membrane is prepared by filling voids of porous materials with the polymer matrix solution. The nanofiber composite membranes are frequently prepared using this route. These methods have their own advantages; however, the precise control of the preparation process and the preparation of ultrathin composite membranes still remain major challenges for them. However, the membrane size and thickness can only be controlled by the volume of the casting solution roughly.

Recently, the direct membrane formation method has been reported for simplifying and optimizing the fabrication process of MEAs. Klingele et al. [30] directly deposited a Nafion^®^ dispersion onto gas diffusion electrodes with catalyst layers as membrane layers, and then pressed two electrodes together with the membrane layers facing each other. This approach constructed the relatively thinner PEM in MEAs to strongly decrease the contact resistance of the membrane and the proton conducting phase of the catalyst layer. Their directly deposited MEAs demonstrated a high power density up to 4.07 W/cm^2^ under H_2_/O_2_ single cell performance test. Breitwieser et al. [31] presented a novel method of MEA preparation by combining scalable deposition and electrospinning to achieve the manufacturing of MEAs with a controlled 3D design; the fabricated composite membranes showed an ultra-thin thickness of 12 μm. These studies demonstrated deposition can improve the freedom degrees of complex MEAs design.

**Figure 1:**
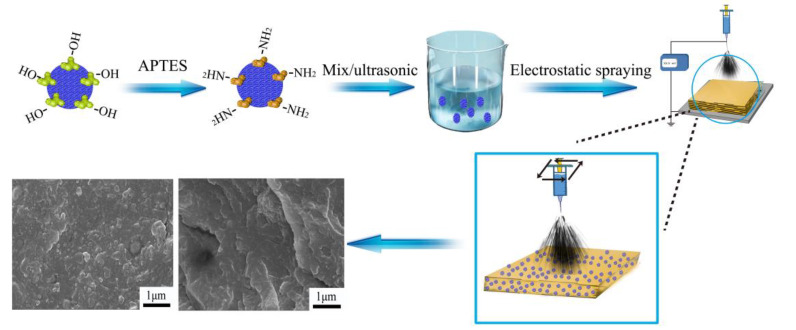
The schematic workflow of the preparation of the PEM.

In this work, we present a novel membrane preparation technology of direct electrostatic deposition (DED), where the membrane is directly prepared on a substrate via electrostatic spraying, which is similar to the electrospinning technology. In this process, the polymer solution was sprayed onto a substrate via a spinneret which has a hole diameter of 0.1 mm. With the solution solidified layer by layer under high-temperature treatment of a substrate, the thickness of the membrane increased at a very slow rate; then the robust and continuous membrane formed. Depending on the increase in thickness on the nanometer scale, the thickness of the membrane can be controlled precisely and simply by spraying time and spraying rate. Besides, the size of the membrane can be precisely controlled by the operation track. Considering the weak proton conductivity of pure SiO_2_ nanospheres, amino groups (–NH_2_) were introduced on their surface to improve compatibility and conduction. More importantly, amino groups in nanomaterials and acid groups in the matrix can form acid–base pairs to accelerate proton transfer. Therefore, we introduced amino-modified SiO_2_ nanoparticles (SiO_2_–NH_2_) into Nafion to prepare the hybrid PEM by DED. The schematic workflow of the preparation of the PEM is shown in Figure 1. Furthermore, the DMFC single cell performance of the as-prepared membrane and Nafion membrane was investigated.

## 2. Materials and Methods

### 2.1. Preparation of the Hybrid Membrane

The detailed synthetic method of SiO_2_–NH_2_ referred to the published literature [32]. Ethanol was chosen as the solvent due to its low boiling point advantage. A certain amount of SiO_2_–NH_2_ nanoparticles and Nafion solution (5%) were successively dispersed in ethanol to obtain a silica/Nafion suspension. Herein, the percent of silica and Nafion in suspension was 0.15% and 2.5%, respectively, and the total fraction of SiO_2_–NH_2_ in the final membrane without solvent was approximately 5.7%. Then the final suspension with Nafion and SiO_2_–NH_2_ underwent ultrasonic treatment for 2 h to break the aggregates. For electrospraying, an electrostatic painting instrument equipped with a solution extrusion device, liquid injection needle, voltage system and heating metal collector was used. The process parameters of hybrid membrane preparation were 1 kV voltage, a tip collector distance of 2 cm, an operation track of 5 cm × 5 cm, a spray rate of 0.1 and 0.15 mL/min and a collector temperature of 75 °C. To compare the single cell performances in DMFC, the prepared hybrid membrane with a thickness of 30 μm was prepared and designed as Nafion/SiO_2_-–NH_2_ in this work. All membranes were impregnated in 2 M H_2_SO_4_ for 12 h and washed with deionized water until neutralized.

### 2.2. Characterization

Scanning electron microscopy (SEM, Hitachi S-4800) and transmission electron microscopy (TEM, JEM 2200FS) were used to observe the morphologies of samples. Energy-dispersive X-ray spectra (EDS) mapping and X-ray photoelectron spectroscopy (XPS) were used to examine the composition of SiO_2_–NH_2_. Wide-angle X-ray diffractometry (XRD) and small-angle X-ray scattering (SAXS) measurements were carried out using an X-ray diffractometer (Rigaku SmartLab SE, Japan) and an Anton Paar SAXS system (SAXS ess mc2, Austria), respectively.

Proton conductivity (*σ*) was measured by AC impedance spectroscopy using an electrochemical workstation under a heated water bath. The frequency range from 0.1 to 10^5^ Hz *σ* was calculated using the following equation:

*σ* = *L*/(*R* · *A*) (1)

where *L*, *R*, and *A* are the electrode distance, the impedance, and the membrane cross-sectional area, respectively.

The methanol permeability was measured via a diffusion cell containing two glass compartments sandwiching the test sample. The methanol permeability was calculated through the following equation:

(2) $$DK=\frac{L\;\cdot V_{B}\;\cdot C_{B\left(t\right)}}{A\cdot C_{A\left(t-t_{0}\right)}}$$

where *DK* is the methanol permeability; *L*, *A*, and *V_B_* correspond to the thickness of the membrane, the effective area, and the volume of the water side, respectively; *C_A_* and *C_B_* are the concentration of methanol (M) in the A side and B side, which can be monitored by gas chromatography (Agilent 7820); $t-t_{0}$ is the test time.

The MEA was prepared by (i) spraying the anode catalyst (PtRu/C, Pt:Ru = 1:1, Johnson Matthey) and cathode catalyst (Pt/C, 60% Pt, Johnson Matthey, London, UK) on the PEM layer (2 cm × 2 cm), and both the catalyst loading was 1 mg/cm^2^; (ii) Sandwiching the above membrane with gas diffusion layers and hot pressing at 100 °C. The DMFC performances of membrane electrode assemblies (MEAs) with different membranes were characterized by polarization curves in a fuel cell testing station (Model TEID160-1NBNNS, Arbin Inc., College Station, TX, USA) at 40 °C. The aqueous methanol (2 M) and oxygen were fed to the anode and cathode at 2 mL/min and 500 mL/min, respectively.

## 3. Results and Discussion

### 3.1. Characterization of SiO_2_–NH_2_

**Figure 2:**
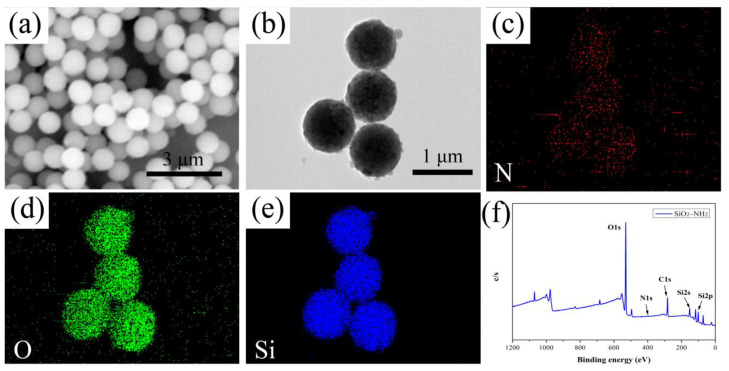
(**a**) SEM, (**b**–**e**) TEM-EDS mapping and (**f**) XPS images of SiO_2_–NH_2_.

SEM, TEM-EDS mapping and XPS tests were used to characterize the morphology and elemental composition of SiO_2_–NH_2_. It could be seen in Figure 2a that the SiO_2_–NH_2_ we synthesized showed a well-defined spherical appearance, and possessed a rough surface caused by the aggregation of –NH_2_. As shown in Figure 2b, N, O and Si elements are uniformly distributed in the nanoparticles, which demonstrates the successful synthesis of the SiO_2_–NH_2_. In addition, the peaks of O (1s), N (1s), C (1s), Si (2s) and Si (2p) shown in Figure 2c could further prove the successful preparation of SiO_2_–NH_2_. Furthermore, the elemental analysis of SiO_2_–NH_2_ by XPS confirmed the Nitrogen percentage of 1.45%, which corresponds to 1.66% of–NH_2_. 

### 3.2. Characterization of Nafion/SiO_2_–NH_2_

The realization of DED via electrostatic spraying mainly depends on the electric force and high-temperature solidification. The membrane fabrication process can be divided into two stages. In the first stage, the surface tension and viscoelastic force of membrane solution are overcome by the electric force, and then spraying type jets are formed and deposited on the collector. Different from the electrospinning process, solvent evaporation during spraying is extremely slow due to the short distance and relatively low voltage. In the second stage, the solution deposited on the collector solidifies to the membrane rapidly because of the high temperature.

**Figure 3:**
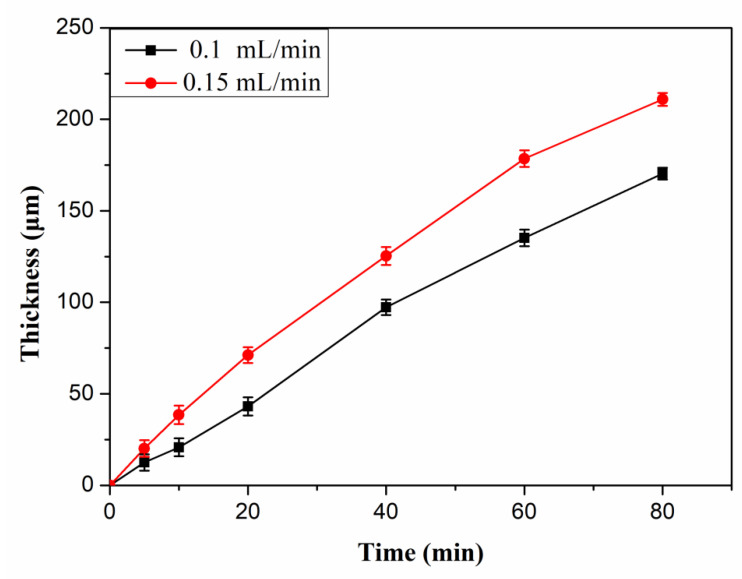
The thickness-time relationship at spraying rates of 0.1 and 0.15 mL/min.

The thickness of the composite membranes changed with the spraying time and spraying rate as shown in Figure 3. The thickness of the membrane shows a linearly increasing trend with the increased spraying time. Moreover, the thickness of the membrane prepared by a spraying rate of 0.15 mL/min is larger than that of the membrane with a spraying rate of 0.1 mL/min. In particular, the error bars of membrane thickness is quite small. Therefore, a membrane with a certain thickness and size can be prepared on a large scale using DED. The above phenomenon shows that the thickness of the membrane prepared by DED can be precisely controlled by spraying time and spraying rate.

**Figure 4:**
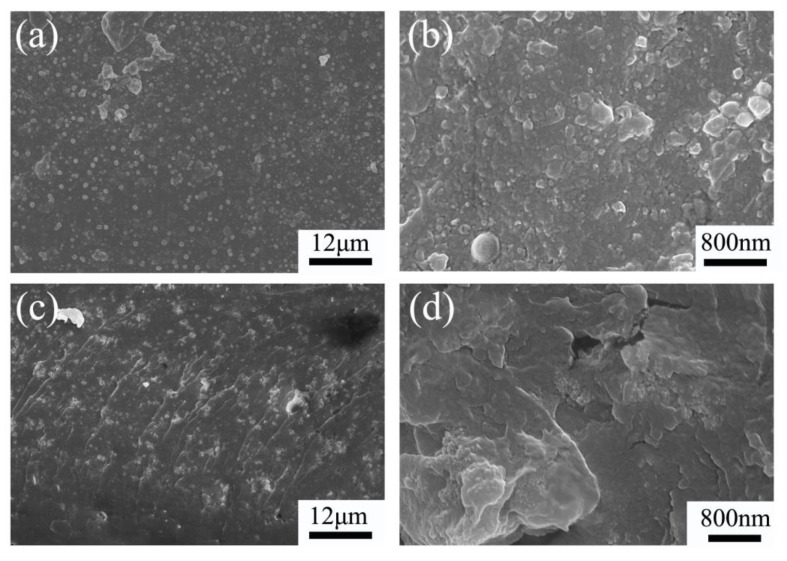
(**a**,**b**) the surface and (**c**,**d**) cross-sectional SEM images of hybrid membranes at different magnifications.

Morphology of the Nafion/SiO_2_–NH_2_: SEM images of the surface and cross-sectional hybrid membranes at different magnifications are shown in Figure 4. As shown in Figure 4, the surface and cross-section of Nafion/SiO_2_–NH_2_ is compact, and no significant crack is shown in the membrane. Furthermore, the SiO_2_–NH_2_ nanoparticles could be clearly observed at both their surface and cross-section. This result revealed the good dispersion of SiO_2_–NH_2_ in the Nafion matrix.

**Figure 5:**
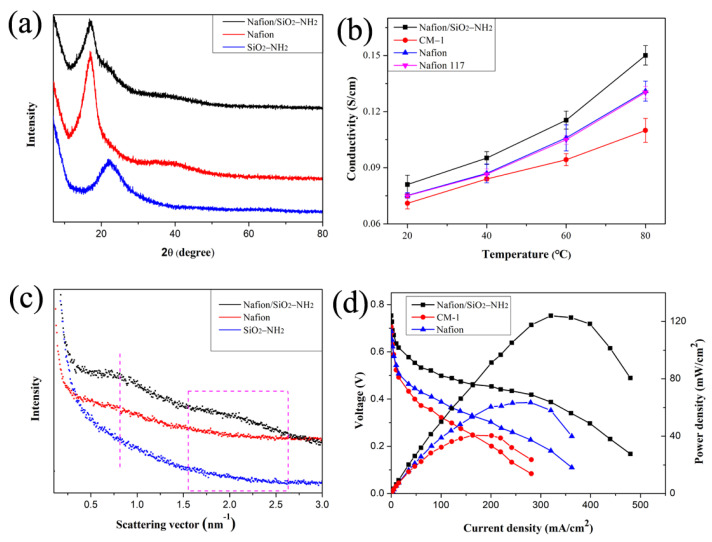
(**a**) XRD curves of Nafion, Nafion/SiO_2_–NH_2_ and SiO_2_–NH_2_; (**b**) Proton conductivity curve, (**c**) SAXS curves and (**d**) DMFC performance (operated at 40 °C and 100% RH) of Nafion/SiO_2_–NH_2_, Nafion and CM-1.

Figure 5a shows the XRD patterns of Nafion, Nafion/SiO_2_–NH_2_ and SiO_2_–NH_2_. All the samples showed amorphous peaks, indicating amorphous characteristics. Comparing the XRD patterns of Nafion/SiO_2_–NH_2_ with Nafion, a new broad peak appeared for the composite membranes at 24°. This result is caused by the redistribution of SiO_2_–NH_2_ in the Nafion matrix and reveals the good compatibility of these components.

The proton conductivity of commercial Nafion 117, pure Nafion and hybrid Nafion membrane prepared by DED is shown in Figure 5b. Pure Nafion exhibited similar proton conductivity with Nafion 117 indicating the processing reliability of DED in membrane formation. The compared membrane containing the same content of SiO_2_–NH_2_ and Nafion matrix are prepared by the casting method and named CM-1. It is interesting that CM-1 showed lower proton conductivity than Nafion/SiO_2_–NH_2_ and Nafion. Nevertheless, Nafion/SiO_2_–NH_2_ exhibited the highest proton conductivity of 0.15 S/cm at 80 °C. This difference in proton conductivity originated from the different microstructure; better distribution of SiO_2_–NH_2_ in a hybrid membrane could bridge ionic clusters in the membrane to form continuous proton transferred channels [33]. During the casting process, nanospheres tend to be distributed on one side of the membrane, affected by gravity. However, high temperature facilitated the micro-volume polymer solution spinneret from solidification on the collector and then formed a layer-by-layer membrane with a homogeneous nanocomposite structure. This conclusion could be verified by the results of cross-sectional SEM images of hybrid membranes. To better verify the above explanation, SAXS of all membranes were characterized (Figure 5c). Nafion/SiO_2_–NH_2_ showed an obvious matrix segment peak and ionomer peak at lower and higher q, respectively. However, the peaks of CM-1 and pure Nafion were not obvious. Based on Bragg’s law, the distance between neighboring ionic clusters in Nafion/SiO_2_–NH_2_ was smaller than in other membranes [34]. Such an observation is also consistent with the proton conductivity results.

Table 1 shows the methanol permeability of Nafion, Nafion/SiO_2_–NH_2_, and CM-1. Compared with the Nafion membrane, Nafion/SiO_2_–NH_2_ exhibited lower methanol permeability, indicating that the introduction of SiO_2_ improves the methanol barrier properties. However, the methanol permeability of CM-1 is lower compared to Nafion/SiO_2_–NH_2_, probably because of the reunion distribution of inorganic particles on one side of the membrane to form methanol barrier layers. This result is consistent with the proton conductivity results.

The polarization and performance curves of passive DMFCs based on pure Nafion and hybrid Nafion/SiO_2_–NH_2_ membranes were collected at 40 °C and 100% RH and are shown in Figure 5c. It can be seen from Figure 5c that the DMFC performance of Nafion/SiO_2_–NH_2_ is enhanced compared to that of Nafion. Particularly, Nafion/SiO_2_–NH_2_ had a maximum power density output of 124.01 mW/cm^2^. However, Nafion and CM-1 only showed maximum power density values of 63.50 and 40.60 mW/cm^2^, respectively. This result is likely due to the following aspects: (i) the ultrathin Nafion/SiO_2_–NH_2_ can transport protons effectively through the membrane; (ii) the well-distributed inorganic silica may improve the water retention and methanol permeability of Nafion; (iii) the more ionic clusters in Nafion/SiO_2_–NH_2_ can provide massive proton transfer sites.

Some published works related to inorganic/organic hybrid membranes were cited for comparison with proton conductivity and power density, as shown in Table 2. The proton conductivity and power density for Nafion/SiO_2_–NH_2_ showed a competitive overall performance than other membranes, verifying that DED is a good application prospect in PEM preparation.

## 4. Conclusions

A novel approach to precisely control the fabrication of PEMs for DMFCs operating was presented in this work. Nafion/SiO_2_–NH_2_ was directly formed on a metal collector enabling the fast, simple and precise fabrication of 30 μm thin composite membranes. Nafion/SiO_2_–NH_2_ showed a maximum power density of 124.01 mW/cm^2^ at 40 °C and 100% RH, which was 95.29% higher than that of Nafion. The results proved that the DED can be a potential method for the precise production of cost-effective and ultrathin membranes.

## Figures and Tables

**Table 1:** The methanol permeability of Nafion, Nafion/SiO_2_–NH_2_, and CM-1.

Samples	Methanol Permeability (10^−7^ cm^2^ s^−1^)
Nafion	17.5
Nafion/SiO_2_–NH_2_	9.8
CM-1	9.1

**Table 2:** Comparison of proton conductivity and power density with other reported PEMs.

PEMs	Proton Conductivity (S/cm)	Power Density (mW/cm^2^)	Ref.
PSU/mMOF/Si-SO_3_H	0.017 (70 °C, 100% RH)	40.8 (70 °C)	[18]
SPEEK/TiNFs-1.0	0.037 (80 °C, 100% RH)	431.5 (60 °C)	[35]
SPEEK/S-SiO_2_/MOF-5	0.00369 (30 °C, 100% RH)	NA	[36]
Nafion/SPES/SiO_2_–3%	0.23 (80 °C, 100% RH)	77.22 (80 °C)	[37]
Nafion/SiO_2_–NH_2_	0.15 (80 °C, 100% RH)	124.01 (40 °C)	This work

## Data Availability

The data presented in this study are available on request from the corresponding author.
